# Editorial: Detrimental effects of hypoxia on brain and cognitive functions

**DOI:** 10.3389/fcogn.2025.1750627

**Published:** 2025-12-19

**Authors:** Alberto Zani, Stephanie Otto, Terry McMorris

**Affiliations:** 1 School of Psychology, Vita-Salute San Raffaele University, Milan, Italy; 2 Naval Medical Research Unit Dayton, Dayton, OH, United States; 3 Department of Sport and Exercise Sciences, University of Chichester, Chichester, United Kingdom

**Keywords:** hypoxia, embodied cognition, mountaineering, aviation, breathing and healthcare throughout the cycle of life, environmental pollution, psychomotor skills and performance, EEG/ERPs and brain metabolism

**Figure 1:**
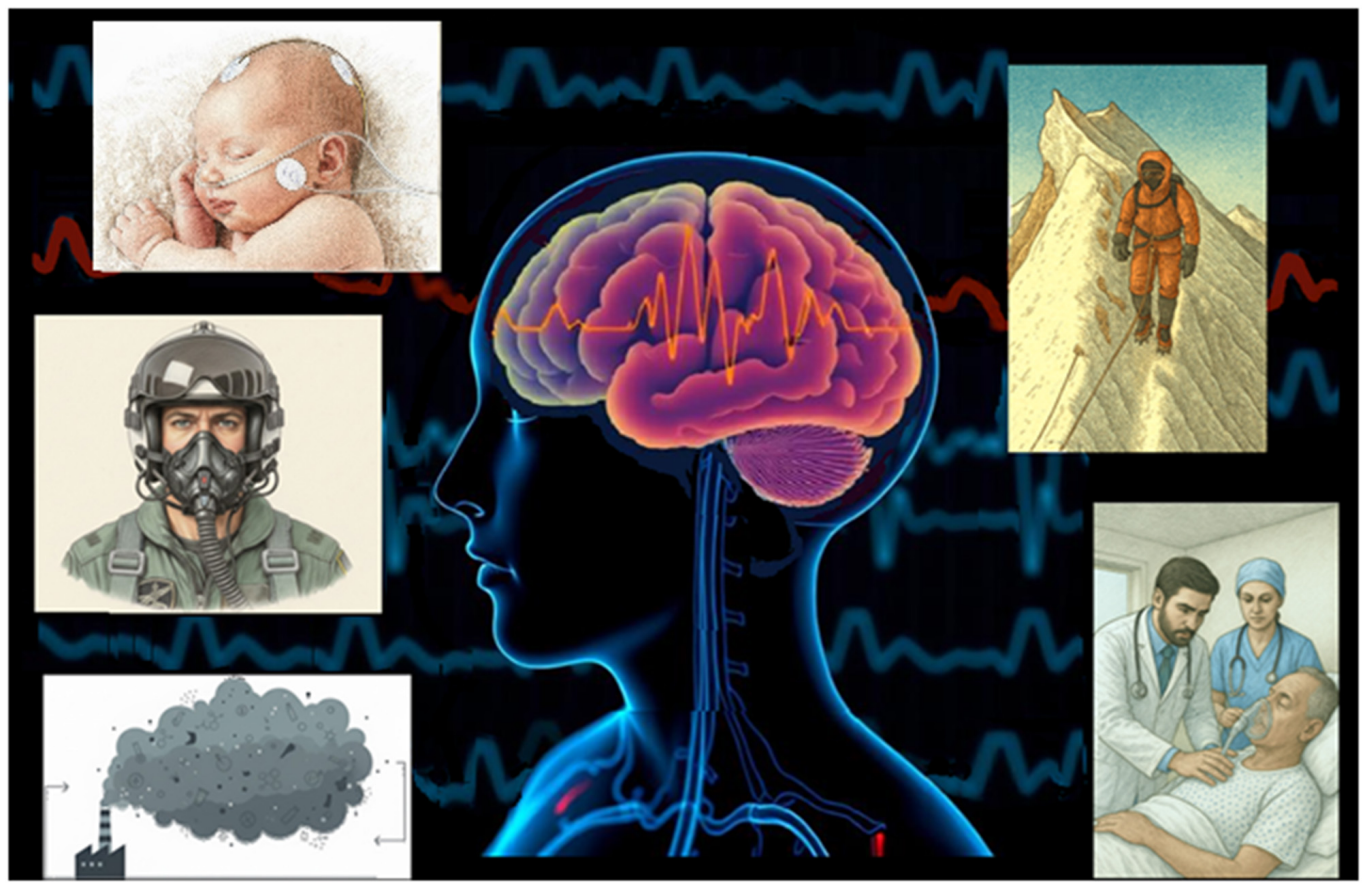
This figure highlights how brain metabolism, neuroelectromagnetic and EEG techniques can assess hypoxia across diverse contexts, including neonatal care, aviation, mountaineering, medical diagnostics, and environmental monitoring.

Breathing connects the body, mind, and environment, essential for somatic and cognitive functions, awareness, and performance. Hypoxia—oxygen deficiency—impairs embodied cognition by reducing cerebral oxygenation (rSO_2_), affecting brain metabolism and EEG/ERP activity related to attention (Altbäcker et al., 2019; Blacker et al., 2021; Zani et al., 2020, 2023; Zani et al.; Borden et al.; Otto et al.), memory (Wang et al., 2022), and executive functions (McMorris et al., 2017; Borden et al.; Zani et al.). It may induce functional and emotional disorders (Kumari et al., 2018; Huang et al., 2026), and damage the neonatal brain (Shabani and Proverbio). In adults, hypoxia—common in polluted environments, high altitude (Zani et al.; Borden et al.), or anesthesia (Chen et al.; Schnaubelt et al.)—impairs neurocognition and can cause neural injury (Minamoto et al., 2024; Zani et al.). Preventing hypoxia is therefore vital in mountaineering, healthcare, aviation, and environmental contexts (see Figure 1).

Reinforcing the importance of preventing hypoxia, Chen et al. studied how permissive hypercapnia (PH) affects cerebral oxygenation and early cognitive function in elderly laparoscopic surgery patients. Analyzing data from 450 patients, they found that PH significantly increased intraoperative cerebral oxygen saturation (rSO_2_), particularly after pneumoperitoneum was established. Patients receiving PH showed less cognitive decline on the first post-operative day, with scores returning to baseline by day 14, outperforming those on conventional ventilation. Importantly, secondary outcomes like recovery time and vital signs showed no adverse differences, indicating that PH is safe. The study suggests moderate hypercapnia may serve as a neuroprotective strategy during surgery by improving cerebral blood flow and oxygenation, thereby reducing early post-operative cognitive impairments. However, given its retrospective focus on early outcomes, further research is needed to confirm benefits and assess long-term effects.

Furthermore, Schnaubelt et al. conducted a prospective study on the prognostic value of near-infrared spectroscopy (NIRS) monitoring of cerebral oxygen saturation (rSO_2_) in out-of-hospital cardiac arrest (OHCA) patients. Measurements taken pre-hospital and in-hospital over up to 72 h showed that higher initial rSO_2_ levels and increasing trends within the first 10 min post-ROSC (i.e., return of spontaneous circulation) were strongly linked to favorable neurological outcomes. An initial rSO_2_ cutoff of around 62% demonstrated good predictive accuracy. The study suggests that early NIRS measurements and dynamic changes may outperform extended Intensive Care Unit (ICU) monitoring for prognosis. These results support NIRS as a valuable, non-invasive bedside tool in post-resuscitation care. Nonetheless, larger studies are still needed to establish standard cutoff values and support clinical adoption.

Shabani and Proverbio report a review of the literature into the effects of Neonatal Hypoxic-Ischemic Encephalopathy (NHIE), the reduced blood and oxygen flow to the brain during pregnancy or birth, which often results in significant brain damage. It is one of the most common complications during childbirth and, as the authors point out, it can have negative lifelong effects on the child. These may include complications such as cerebral palsy, cognitive and learning impairment and emotional difficulties. Structural anomalies, such as reduced corpus callosum volume, reduced white matter integrity, decreased brainstem volume and increased ventricular size are also found. Furthermore, it can result in autism, and other disorders. The standard treatment, therapeutic hypothermia (TH) can have positive effects even when NHIE is severe.

Nevertheless, NHIE survivors face life with increased risk of cognitive impairments, including deficits in processing speed, problem-solving, and attention. They also often show poor psychomotor skills. Common psychiatric deficits include mood and personality disorders, aggressiveness, anxiety, expression, autism, attention deficit hyperactivity disorder and schizophrenia.

Zani et al. present a comprehensive review of the literature covering neurocognitive effects of hypoxia in various environmental conditions. Moreover, they examine similarities and differences concerning effects of normobaric hypoxia and hypobaric hypoxia. The authors review effects on neuroimaging markers, such as electroencephalogram (EEG), event related potentials (ERPs), positron emission tomography (PET) and functional magnetic resonance imaging (fMRI). The types of tasks examined are cognitive and motor functions related to short-term memory, attention and executive control. Amplitudes of ERPs P3b, ADAN, and CNV increased with cognitive demands in hypoxia, indicating a compensatory response. Furthermore, integrations of sensory and cognitive functions are examined using measures of delta, gamma, theta, and alpha oscillatory systems. Despite this, the authors conclude that the tasks most affected are executive functions and short-term memory recall. Additionally, they highlight that visual and audio-spatial skills are also negatively impacted.

The research paper by Borden et al. provides valuable insights into the effects of hypoxia on cognitive and neural functions relevant to tactical aviation. The study extends previous work by using a more ecologically valid hypoxia simulation device, the ODHT, or “On-Demand Hypoxia Trainer,” which closely mimics in-flight conditions. Results demonstrate that physiological responses such as SpO_2_ decline and increased heart rate occur rapidly within minutes of exposure, while subjective symptom reporting is delayed, highlighting limitations of self-awareness for hypoxia detection. Importantly, cognitive performance deteriorates notably in complex tasks involving visual search, working memory, and inhibitory control, especially at higher altitudes. Neural markers, specifically the MMN/P3a ERP complex, show reductions in amplitude associated with sensory processing deficits, aligning with physiological and behavioral impairments. The findings underscore the potential for EEG-based neural markers to serve as objective indicators of hypoxia-induced impairment, advancing the development of real-time monitoring systems to enhance pilot safety.

Otto's et al. review builds on Borden et al. to explore acute hypoxia's neural effects via EEG. It highlights how hypoxia, common in high-altitude aviation and mountaineering, impairs cognition, with EEG spectral power and ERPs as sensitive markers. Hypoxia increases delta, theta, and beta power, while alpha responses vary. ERPs like P3a and mismatch negativity are reliably affected, indicating deficits in attention and sensory processing. The review also discusses emerging EEG technologies, including dry and ear-EEG systems, for real-time operational monitoring to improve pilot safety. Challenges include individual variability, methodological differences, and EEG's limited spatial resolution. The authors call for further research on long-term effects, neural mechanisms, and multimodal imaging. Overall, the review positively advocates for EEG's role in hypoxia research and in-flight monitoring to mitigate risks in aviation and extreme environments.

Within cognitive neuroscience, this Research Topic emphasizes the harmful consequences of hypoxia across various domains, including embodied psychomotor performance, mountaineering, medicine, aviation, and ecology.
